# The Course of Asthma in Young Adults: A Population-Based Nine-Year Follow-Up on Asthma Remission and Control

**DOI:** 10.1371/journal.pone.0086956

**Published:** 2014-01-29

**Authors:** Lucia Cazzoletti, Angelo Guido Corsico, Federica Albicini, Eti Maria Giulia Di Vincenzo, Erica Gini, Amelia Grosso, Vanessa Ronzoni, Massimiliano Bugiani, Pietro Pirina, Isa Cerveri

**Affiliations:** 1 Unit of Epidemiology and Medical Statistics, University of Verona, Verona, Italy; 2 Department of Molecular Medicine, University of Pavia, Pavia, Italy; 3 Division of Respiratory Diseases, Istituto di Ricovero e Cura a Carattere Scientifico “San Matteo” Hospital Foundation, University of Pavia, Pavia, Italy; 4 Unit of Respiratory Medicine, National Health Service, Turin, Italy; 5 Institute of Respiratory Diseases, University of Sassari, Sassari, Italy; Universität Bochum, Germany

## Abstract

**Background:**

Only few longitudinal studies on the course of asthma among adults have been carried out.

**Objective:**

The aim of the present prospective study, carried out between 2000 and 2009 in Italy, is to assess asthma remission and control in adults with asthma, as well as their determinants.

**Methods:**

All the subjects with current asthma (21–47 years) identified in 2000 in the Italian Study on Asthma in Young Adults in 6 Italian centres were followed up. Asthma remission was assessed at follow-up in 2008–2009 (n = 214), asthma control at baseline and follow-up. Asthma remission and control were related to potential determinants by a binomial logistic and a multinomial logistic model. Separate models for remission were used for men and women.

**Results:**

The estimate of the proportion of subjects who were in remission was 29.7% (95%CI: 14.4%;44.9%). Men who were not under control at baseline had a very low probability of being in remission at follow-up (OR = 0.06; 95%CI:0.01;0.33) when compared to women (OR = 0.40; 95%CI:0.17;0.94). The estimates of the proportion of subjects who were under control, partial control or who were not under control in our sample were 26.3% (95%CI: 21.2;31.3%), 51.6% (95%CI: 44.6;58.7%) and 22.1% (95%CI: 16.6;27.6%), respectively. Female gender, increasing age, the presence of chronic cough and phlegm and partial or absent asthma control at baseline increased the risk of uncontrolled asthma at follow-up.

**Conclusion:**

Asthma remission was achieved in nearly 1/3 of the subjects with active asthma in the Italian adult population, whereas the proportion of the subjects with controlled asthma among the remaining subjects was still low.

## Introduction

Asthma is a major chronic respiratory disease [1] whose prevalence is still on the increase in Italy [2]. Unlike most chronic diseases, which start in adulthood and tend to progress over time, asthma may develop at any age and remittance is relatively common [3], especially during adolescence [4]. However, only few studies on the course of asthma among adults [5]–[7] have been carried out.

Progress in asthma treatment has reduced the symptoms of asthma and the frequency of severe life–threatening exacerbations of the disease. Asthma is now a treatable disease, but it is not curable. At present, most discussions on asthma treatment goals revolve around disease control [8], [9], and little attention is paid to remission as an achievable therapeutic goal [10].

Detecting the factors related to asthma remission or control could have clinical and public health implications. Few longitudinal studies have focused on these implications and many of them have been limited to short follow-up studies [11], [12], [6].

The aim of the present prospective study, carried out between 2000 and 2009 in Italy, was to assess asthma remission and control in adult subjects with asthma and their determinants.

## Methods

### Study design

A screening questionnaire investigating the presence of respiratory symptoms and asthma management was sent to random samples of people aged 20–44 years at the time of recruitment, in the frame of the Italian Study on Asthma in Young Adults (ISAYA) carried out between 1998 and 2000 [5], [13].

All the subjects in the 6 Italian centres (Verona, Torino, Pavia, Sassuolo, Pisa, Sassari), who reported current asthma at the screening questionnaire (self-reported physician's diagnosis of asthma and at least one asthma attack in the last 12 months and/or current use of anti-asthma drugs), between April and June 2000, were invited to participate in a phone interview aimed at collecting information on symptoms and treatment [14]. Out of the 458 eligible subjects with current asthma at the postal survey, 354 (77.3%) subjects participated in the telephone survey. These subjects were re-surveyed by telephone or in a face to face interview between 2008 and 2009, and 214 subjects (response rate: 60.4%) participated in the follow-up.

### Ethics approval

Local ethics committees at each center approved the local study protocols and the follow-up study was approved by the ethic committee of the University of Pavia.

### Definition of asthma remission

Remission was defined as no current use of asthma medication, no asthma-like symptoms (wheezing, tightness in the chest, shortness of breath) and no asthma attacks in the last 12 months, as reported by the subjects at follow-up.

### Definition of asthma control

Asthma control was defined by using a customized version of the asthma control definition provided in the Global Initiative for Asthma (GINA) guidelines [15], [16]. The definition of asthma control was mainly based on the frequency of diurnal and nocturnal symptoms, the limitations in daily activities and the need for reliever/rescue treatment. According to this definition, asthma was considered to be controlled if all the following features were present: diurnal symptoms less than once a week and no asthma attacks in the last 3 months, no activity limitations in the last 12 months, no nocturnal symptoms in the last 3 months, short-acting β_2_-agonists twice or less per week in the last 3 months and no use of oral steroids in the last 12 months. Asthma was considered to be partly controlled if 1 or 2 of the above features were absent. Asthma was considered to be uncontrolled if more than 2 features were absent or if asthma, shortness of breath, or wheezing had caused hospital/emergency department admissions in the last 12 months; oral steroids were used in short courses or continuously in the last 3 months; or the subject had more than 12 asthma attacks in the last 3 months.

Information on lung function was not available.

### Classification of treatment

Treatment was classified in one of five steps according to the average daily dose of inhaled corticosteroids (ICS) based on the last 3 months [17]:

**STEP 1:** As needed short-acting β_2_-agonist;

**STEP 2:** Sustained release theophylline OR Leukotriene modifier OR budesonide <400 μg (low-dose) OR an equipotent dose of other ICS;

**STEP 3:** Budesonide < = 400 μg or an equipotent dose of other ICS plus long-acting β_2_-agonist OR

Budesonide < = 400 μg or an equipotent dose of other ICS plus leukotriene modifier OR

Budesonide < = 400 μg or an equipotent dose of other ICS plus sustained release theophylline OR

Budesonide >400 μg (medium or high dose) or an equipotent dose of other ICS;

**STEP 4:** Budesonide >400 μg or an equipotent dose of other ICS plus long-acting β_2_-agonist OR

Budesonide >400 μg or an equipotent dose of other ICS plus leukotriene modifier OR

Budesonide >400 μg or an equipotent dose of other ICS plus sustained release theophylline;

**STEP 5:** Oral corticosteroids or Anti-IgE treatment

The equipotent doses of other ICS – beclomethasone dipropionate, flunisolide, fluticasone, mometasone, ciclesonide and triamcinolone – were the ones reported in the GINA guidelines [17].

### Statistical analysis

Data were summarized as means with standard deviations (s.d.), or as medians with interquartile ranges (IQRs), when appropriate, for continuous variables and as percentages for categorical variables. Comparison of variables across strata was performed by using the χ^2^ test for categorical variables and the t-test or the Wilcoxon test for continuous variables. The conventional 5% level of statistical significance was used.

We addressed the problem of unit non-response by the assignment of statistical weights to the participants [18], [19]. First, we fitted a logistic stepwise regression model (backward approach) to all the participants at baseline, the resulting fitted model was then used to predict a response propensity for each respondent, and the inverse of the predicted response propensity was the nonresponse adjustment associated with each respondent. The significance level for removal from the stepwise model was 0.20 and the variables included in the final model were smoking habits, asthma attacks in the last 12 months, allergic rhinitis and hospital or emergency department admissions because of asthma, shortness of breath, or wheezing in the last 12 months. Age, gender, age at asthma onset, current asthma drug use, asthma-like symptoms in the last 12 months, chronic cough and phlegm, activity limitations in the last 12 months were excluded from the final model. We trimmed the upper 5% of our weights to control for a possible large variation of the weights (to 2.233) [20], [21].

To identify the factors associated with asthma remission, a logistic regression model was fitted to the data, using a dummy indicator as the dependent variable (presence/absence of remission at follow-up). The logistic regression model was weighted by using the mentioned non-response adjustment. We assessed the association between several determinants (gender, smoking habits, allergic rhinitis, age, age at asthma onset, ICS use in the last 3 months) measured at baseline and asthma remission at follow-up, adjusting for the effect of the type of survey (telephone/face to face). Moreover, we assessed the interactions between gender and the other covariates, and the presence of interactions was assessed using the Wald test. The interactions between gender and smoking habits (p for the interaction = 0.023), gender and control at baseline (p = 0.003) and gender and age at asthma onset (p = 0.023) were statistically significant. The complete model included the significant interaction terms, and 2 separate models were used for men and women. Multivariate associations of potential determinants with asthma remission were expressed by odds ratios (OR) and their 95% Confidence Intervals (95%CIs).

To identify the factors associated with asthma control, a multinomial regression model was fitted to the data, using a 3-level dependent variable (controlled, partially controlled and uncontrolled asthma at follow-up). The multinomial regression model was weighted by using the inverse of the predicted response propensity. Multivariate associations of potential determinants with asthma control were expressed by relative risk ratios (RRRs; using controlled asthma as the reference category) and their 95%CIs.

The multivariate models identified centers as clustering factors, and the Huber/White sandwich estimate of variance was used to adjust for within-center correlation [22].

The statistical analyses were performed using STATA software, release 12.0 (StataCorp, College Station, TX, USA).

## Results

### Participants and non participants

Gender, age and smoking habits were not significantly different between subjects who did participate in the follow-up and subjects who did not (Table 1). Participants and non participants did not differ with regard to asthma-like symptoms in the previous 12 months, current asthma drug use, chronic cough and phlegm, activity limitations in the last 12 months and hospital/emergency department admissions because of asthma, shortness of breath, or wheezing in the last 12 months, as reported at baseline. Subjects participating in the follow-up were significantly more likely to report allergic rhinitis (76.2%), with respect to subjects who did not participate (65.5%) (p = 0.029). Furthermore, they were significantly less likely to report asthma attacks in the last 12 months (79.0%), with respect to subjects who did not participate (90.0%) (p = 0.006).

**Table 1:** Main baseline characteristics of the subjects participating and not participating in the follow-up survey.

	Non participants	Participants	All subjects at baseline	p-value
	n = 140	n = 214	n = 354	
***Mean age (years), (sd)***	33.2 (7.0)	34.2 (7.5)	33.8 (7.3)	0.242
***Gender (%)***				0.577
Male	49.3	46.3	47.5	
Female	50.7	53.7	52.5	
***Smoking habits (%)***				0.125
Non smoker	42.9	48.6	46.3	
Ex-smoker	16.4	21.0	19.2	
Current smoker	40.7	30.4	34.5	
***Age at onset of asthma (years), median***	14.0	13.0	14.0	0.738
*Age at onset of asthma (IQR* * *)*	(5.0–25.0)	(6.0–22.0)	(6.0–24.0)	
***Asthma attacks in the last 12 months (%)***	90.0	79.0	83.3	0.006
***Current asthma drug use (%)***	66.4	69.2	68.1	0.590
***Asthma-like symptoms (wheezing, tightness, shortness of breath) in the last 12 months (%)***	84.9	87.8	86.7	0.434
***Chronic cough and phlegm (%)***	33.1	33.3	33.2	0.963
***Allergic rhinitis (%)***	65.5	76.2	71.9	0.029
***Lost work days in the last 12 months (%)***	18.3	14.8	16.2	0.408
***Impaired days in the last 12 months (%)***	22.2	19.4	20.5	0.531
***Hospital or emergency department admissions because of asthma, shortness of breath, or wheezing in the last 12 months (%)***	10.4	5.6	7.4	0.099

* IQR, Interquartile Range: (1^st^ quartile–3^rd^ quartile).

P-values refer to the comparison between non participants and participants.

### Asthma remission and its determinants

Asthma remission could not be defined for 4 subjects at follow-up due to missing information on current asthma drug use, asthma symptoms or attacks in the last 12 months. After adjusting for non-response and for the effect of covariates, the estimate of the proportion of subjects who were in remission was 29.7% (95%CI: 14.4%;44.9%). A regression model for remission at follow-up was estimated separately for men and women (Table 2 and 3). The direction of the association between asthma control at baseline and remission was consistent in men and women, but men who were not under control at baseline had a very low probability of being in remission at follow-up (OR = 0.06; 95%CI:0.01;0.33, Table 2) when compared to women (OR = 0.40; 95%CI:0.17;0.94, Table 3). Age at asthma onset was not significantly associated with remission in men (OR = 1.02; 95%CI:0.96;1.08, Table 2); whereas, in the case of women, as age at onset of the disease increased, the likelihood of being in remission at follow-up increased (OR = 1.07; 95%CI:1.00;1.14, Table 3).

**Table 2:** Main baseline characteristics of the men with non remittent asthma and with remittent asthma at follow-up and adjusted Odds Ratios* (CI95%) for the effects of these variables reported at baseline on asthma remission at follow-up.

	Non remittent asthma	Remittent asthma	Unadjusted p-value	Adjusted OR* (CI95%)
	n = 68	n = 28		
***Age at baseline (years), mean(sd)***	33.4 (7.8)	33.2 (6.9)	0.893	0.95 (0.88,1.02)
***Smoking habits (%)^£^***			0.772	
*Non smoker*	42.6	46.4		1.00
*Ex-smoker*	20.6	14.3		1.18 (0.15;9.25)
*Current smoker*	36.8	39.3		0.85 (0.22;3.25)
***Age at onset of asthma (years), median***	10	10	0.842	1.02 (0.96;1.08)
*Age at onset of asthma (years), (IQR)* §	(6–16)	(5–20)		
***ICS use in the last 3 mo. at baseline (%)***	43.9	11.1	**0.002**	0.36 (0.08;1.58)
***Asthma control at baseline (%)*** ‡			**<0.001**	
*Total control*	23.5	75.0		1.00
*Partial control*	53.0	21.4		**0.16 (0.07;0.62)**
*No control*	23.5	3.6		**0.06 (0.01;0.33)**
***Allergic rhinitis (%)***	82.4	71.4	0.231	0.56 (0.11;2.91)

* Also adjusted for the type of interview (telephone vs face to face).

£ Overall adjusted p-value for smoking  = 0.5896.

§ IQR, Interquartile Range: (1^st^ quartile–3^rd^ quartile).

‡ Overall adjusted p-value for asthma control at baseline <0.0001.

Significant unadjusted p-value and adjusted OR (p<0.05) are reported in bold.

**Table 3:** Main baseline characteristics of the women with non remittent asthma and with remittent asthma at follow-up and adjusted Odds Ratios* (CI95%) for the effects of these variables reported at baseline on asthma remission at follow-up.

	Non remittent asthma	Remittent asthma	Unadjusted p-value	Adjusted OR* (CI95%)
	n = 81	n = 33		
***Age at baseline (years), mean(sd)***	34.6 (6.9)	35.9 (8.5)	0.431	1.00 (0.94;1.06)
***Smoking habits (%)^£^***			0.452	
*Non smoker*	54.3	48.5		1.0
*Ex-smoker*	23.5	18.2		0.39 (0.03;5.66)
*Current smoker*	22.2	33.3		3.82 (0.56;26.27)
***Age at onset of asthma (years), median***	14	19.5	**0.027**	**1.07 (1.00;1.14)**
*Age at onset of asthma (years), (IQR)* §	(5–25)	(12–31.5)		
***ICS use in the last 3 mo. at baseline (%)***	39.2	21.98	0.081	0.53 (0.27;1.06)
***Asthma control at baseline (%)*** ‡			0.425	
*Total control*	31.6	37.5		1.00
*Partial control*	30.4	37.5		0.71 (0.20;2.47)
*No control*	38.0	25.0		**0.40 (0.17;0.94)**
***Allergic rhinitis (%)***	72.8	72.7	0.990	1.49 (0.78;2.85)

* Also adjusted for the type of interview (telephone vs face to face).

£ Overall adjusted p-value for smoking <0.0001.

§ IQR, Interquartile Range: (1^st^ quartile–3^rd^ quartile).

‡ Overall adjusted p-value for asthma control at baseline  = 0.0791.

Significant unadjusted p-value and adjusted OR (p<0.05) are reported in bold.

Smoking was associated with asthma remission in women (p for the overall association<0.0001, Table 3), but not in men (p = 0.5896, Table 2).

### GINA-based asthma control in subjects with active asthma

Sufficient data to define asthma control at baseline were available for 147 subjects with active asthma at follow-up: 41 (27.9%) of them had controlled, 60 (40.8%) partially controlled and 46 (31.3%) uncontrolled asthma.

Asthma control was defined for 138 subjects with active asthma at follow-up: 38 (27.5%) of them had controlled, 69 (50.0%) partially controlled and 31 (22.5%) uncontrolled asthma.

**Figure 1:**
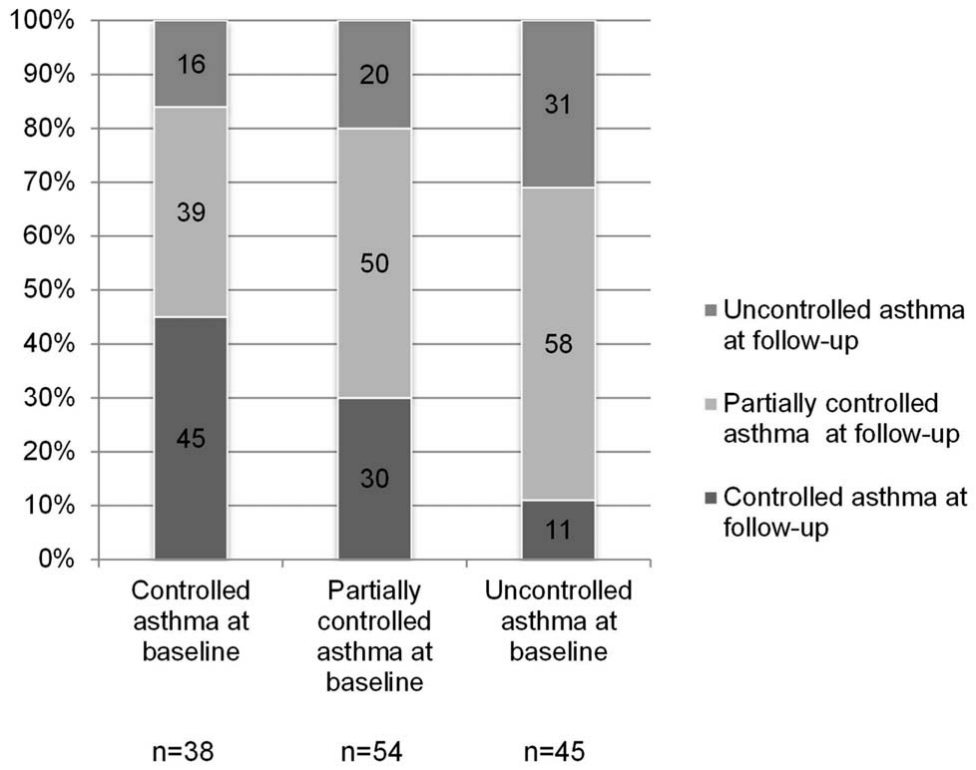
Distribution of GINA-based asthma control at follow-up according to GINA-based asthma control measured at baseline. The percentage of subjects in each category of GINA-based asthma control at follow up is reported inside histograms. The numbers of subjects with controlled, partially controlled and uncontrolled asthma at baseline are different from those reported in the result section due to missing data on control at follow-up.

The distribution of asthma control at follow-up was significantly associated with asthma control at baseline (p = 0.016) (Figure 1): the percentage of subjects with uncontrolled asthma at follow-up was higher for subjects with uncontrolled (31%) or partially controlled (20%) compared to subjects with controlled (16%) asthma at baseline. The percentage of subjects with controlled asthma at follow-up was higher for subjects with controlled (45%) as compared to subjects with partially controlled (30%) or uncontrolled (11%) asthma at baseline. It is noteworthy to mention that for more than 50% of the subjects who had controlled asthma at baseline, asthma was no longer under control at follow-up.

After adjusting for non-response and for the effect of covariates, the estimates of the proportion of subjects who were under control, partial control or who were not under control were 26.3% (95%CI: 21.2%;31.3%), 51.6% (95%CI: 44.6%;58.7%) and 22.1% (95%CI: 16.6%;27.6%), respectively.

### Determinants of asthma control at follow up

At follow-up, women were more likely to have partially controlled (OR = 4.87; 95%CI:2.66,8.93) or uncontrolled asthma (OR = 4.28; 95%CI:1.94,9.42) than controlled asthma with respect to men. Increasing age, the presence of chronic cough and phlegm and partial or absent asthma control at baseline increased the risk of uncontrolled asthma at follow-up (Table 4).

**Table 4:** Adjusted RRRs* (CI95%) for the effects of several variables reported at baseline or at follow-up on asthma control (partial control or no control) at follow-up, in subjects with non remittent asthma (n = 127).

		Partial control		No control	
		RRR	CI95%	RRR	CI95%
***Age (years)***		1.00	0.93;1.08	**1.04**	**1.02;1.05**
***Gender***	Male	1.00		1.00	
	Female	**4.87**	**2.66;8.93**	**4.28**	**1.94;9.42**
***Smoking habits at baseline***	No smoker	1.00		1.00	
	Ex-smoker	0.85	0.14;5.02	1.31	0.32;5.31
	Current smoker	2.40	0.72;7.95	1.64	0.75;3.58
***Age at onset of asthma (years) at baseline***		1.05	0.99;1.11	1.01	0.94;1.09
***Asthma control at baseline***	Total control	1.00		1.00	
	Partial control	1.79	0.81;3.96	**2.66**	**1.00;7.05**
	No control	3.83	1.01;14.51	**7.67**	**3.47;16.95**
***Allergic rhinitis***	No	1.00		1.00	
	Yes	0.57	0.11;3.07	2.85	0.17;46.61
***Chronic cough and phlegm***	No	1.00		1.00	
	Yes	1.35	0.73;2.47	**2.96**	**1.63;5.37**
***ICS use in the last 3 months at baseline***	No	1.00		1.00	
	Yes	4.30	0.79;23.52	2.31	0.35;15.54
***ICS use in the last 3 months at follow-up***	No	1.00		1.00	
	Yes	0.57	0.30;1.06	0.46	0.10;2.08

The reference category is control.

* RRRs were obtained through a multinomial logistic regression model with the controlled group as the reference category. RRRs were also adjusted for the type of interview (telephone vs face to face).

RRRs significantly different from 1 are reported in bold (p<0.05).

### Asthma control and asthma management at follow-up

**Figure 2:**
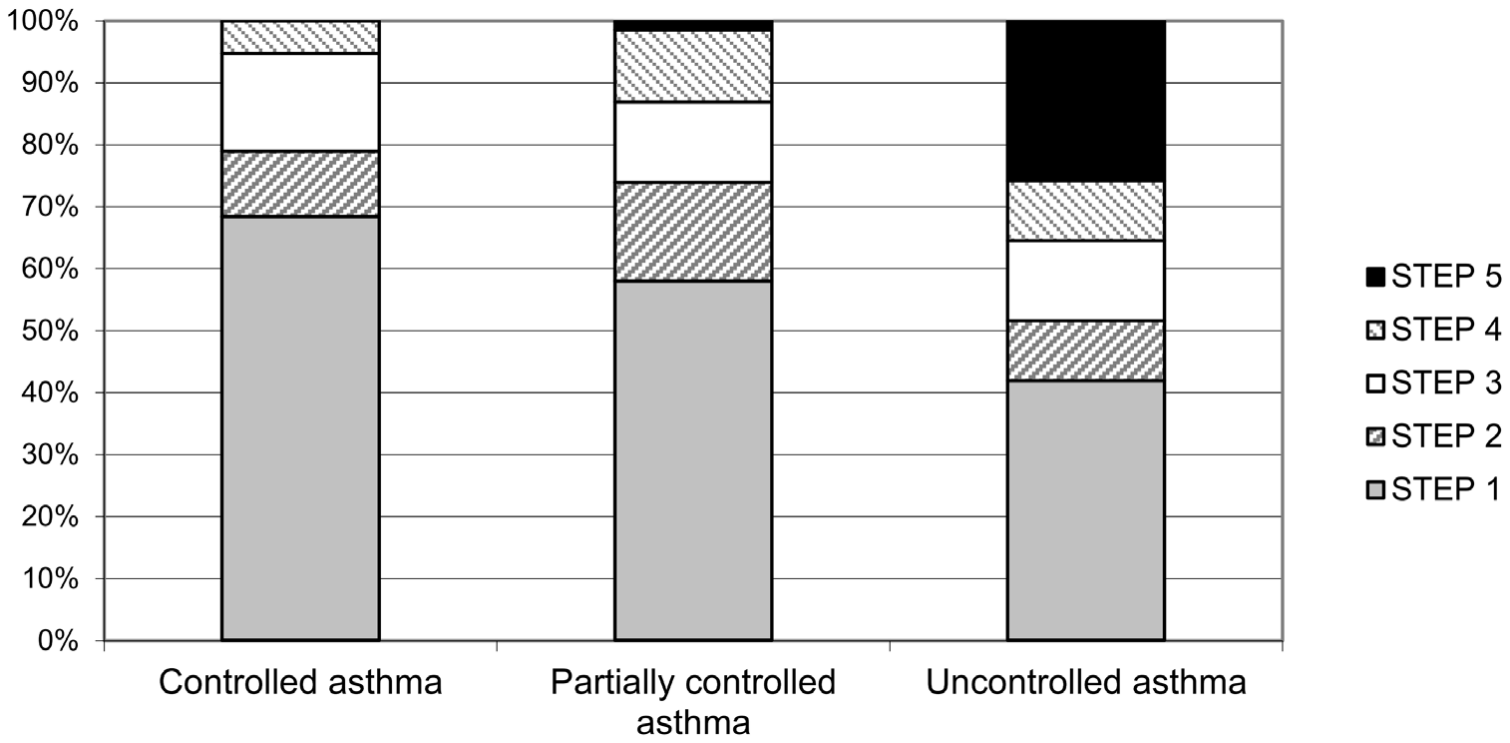
Distribution of treatment step* according to GINA classification in subjects with controlled asthma, partially controlled asthma and uncontrolled asthma, at follow-up. * STEP 1: As needed short-acting β_2_-agonist; STEP 2: Sustained release theophylline OR Leukotriene modifier OR Low-dose Inhaled Corticosteroids (ICS); STEP 3: Low-dose ICS plus long-acting β_2_-agonist OR Low-dose ICS plus leukotriene modifier OR Low-dose ICS plus sustained release theophylline OR Medium or high dose ICS; STEP 4: Medium or high dose ICS plus long-acting β_2_-agonist OR Medium or high dose ICS plus leukotriene modifier OR Medium or high dose ICS plus sustained release theophylline; STEP 5: Oral corticosteroids or Anti-IgE treatment.

At follow-up, the percentage of subjects who were not on controller therapy, i.e. who were using at most short-acting β_2_-agonists when needed, was 68%, 58% and 42% in subjects with controlled, partially controlled and uncontrolled asthma, respectively (Figure 2).

## Discussion

### Asthma remission and its determinants

It has been reported that remission prevalence in follow-up studies in adults with asthma range from 5 to 40%, depending on the period of follow-up and the definition of remission [10]. There are only few population-based longitudinal studies on asthma remission in adults [5]–[7], [11], [12]. In the present prospective longitudinal study, we estimated that nearly 1/3 (29.7%) of the subjects with current asthma sampled from the Italian adult population aged 20–44 years recover from their asthma after about 10 years. In a postal survey with a similar follow-up period and remission definition, Ekerljung and colleagues found that the 10-year remission was 14.6% in subjects with asthma aged 20–69 years [7]. The difference with respect to our study, could be due to the different age range of the studied population, as there is evidence that the remission rate decreases with increasing age [23].

In contrast to other chronic inflammatory diseases, such as rheumatoid arthritis, there is no clear consensus on what constitutes remission of asthma [10]. In most publications, asthma remission is defined as the absence of asthma symptoms without current pharmacological therapy for a prolonged period of time [6], [24]–[26], although normal lung function and no airway hyperresponsiveness are sometimes included in the definition of asthma remission [27]. If the absence of symptoms is central to the definition of remission, it is very important to establish how long the symptoms should be absent. In the present study, we have defined asthma remission as the absence of asthma symptoms and of the use of asthma medicines in the last 12 months. It could be argued that this period is too short to unambiguously identify the occurrence of asthma remission. However, other investigators used the same time frame [7], [11], [12], [25], and a longer period could raise the problem of recall bias. One year without symptoms has been accepted as the minimum, given the frequent seasonal variability of asthma, but the different observation period chosen in other studies can be responsible, at least in part, for the different results [6]. On the other hand, we cannot exclude that the short time interval we took into consideration to assess asthma remission could have led to an overestimation of the proportion of subjects with asthma in remission at follow-up.

To our knowledge, no studies have assessed the relationship between asthma control, as defined by the GINA guidelines and measured at baseline, and asthma remission at follow-up. Previous studies have noted that markers of asthma control like the presence and the frequency of most respiratory symptoms were correlated with the persistence of asthma, suggesting that subjects with a mild form of the disease are more likely to experience asthma remission [6], [11], [12], [23]. In this study, the lack of asthma control as defined by the GINA guidelines at baseline was associated with the persistence of asthma but we found that gender modified this relationship: men who were not under control at baseline had a very low probability of being in remission at follow-up when compared to women.

Previous studies found early onset asthma to be more likely to remit than later onset asthma [28], [29]. In our study, we found a differential effect of gender on the association between age at onset and asthma. As the age at onset of the disease increased, the likelihood of women being in remission at follow-up increased.

It is well known that the relationship between smoking habits and asthma remission is not clear. Some evidence has been found that quitting smoking favours the remission of asthma [6], [12]. On the contrary, also a direct association between smoking and remission has been documented and has been associated to the ‘healthy smoker’ effect, meaning that those subjects with more susceptible airways do not take up smoking or quit early [26], [28]. In our study, we found a differential effect of gender on the association between smoking habits and asthma. In particular, smoking was not associated with asthma remission in men, whereas in the case of women the ‘healthy smoker’ effect was manifest.

### Asthma remission and asthma control

Recently, it has been argued that asthma remission rather than control should be regarded as the true goal of treatment [10]. A few studies have considered the natural history and the remission of asthma over very long periods, even as long as 2 or 3 decades or more [23], [24], [26], [28], but the majority of the studies on asthma control were cross-sectional studies [16], [30]–[32] or clinical studies [8] conducted over short time intervals, so the information available on the long-term control of asthma is scarce.

In agreement with previous studies, we found that the subjects with controlled asthma at baseline were more likely to have controlled asthma at follow-up. Combescure et al. have found that patients with severe asthma had difficulty in leaving unacceptable control [33]. Our data, according to the study of Koster et al [34], consider a much longer time interval with respect to this study, and indicate a certain instability in asthma control.

### Determinants of asthma control

It has already been reported in cross-sectional and/or clinical studies that women have less controlled [13], [35] and more severe [36] asthma, greater bronchial responsiveness [37], use more healthcare services [38] and have a poorer health-related quality of life [39] than men with asthma. Our study supplements this evidence by showing that, after adjusting for asthma control at baseline, female gender is an independent predictor of worse asthma control 9 years later.

Current smoking has been found to be associated with uncontrolled asthma [40], but we did not find a significant association between smoking habits at baseline and asthma control nine years later. We found that smoking habits were associated with asthma control at baseline, suggesting a higher level of asthma control in non-smokers compared to ex- and current smokers (data not shown). This is likely to be the reason why, after controlling for asthma control at baseline, smoking is no longer associated with asthma control at follow-up.

In our study, subjects with chronic cough and phlegm at baseline were more likely to have uncontrolled asthma nine years later than subjects without chronic cough and phlegm at baseline, regardless of smoking. A positive association between chronic cough and phlegm and uncontrolled asthma has been previously reported in cross-sectional studies [16], [35].

Rhinitis has been shown to be associated with the lack of asthma control [41]. We did not find a significant association between rhinitis at baseline and asthma control 9 years later, probably due to our incomplete diagnosis of allergic rhinitis.

### Asthma treatment and control

We found that a considerable part of the subjects with partially controlled or uncontrolled asthma at follow-up (58% and 42%, respectively) was not following a therapy aimed at keeping asthma under control: such large proportions of subjects with partially controlled or uncontrolled asthma who were not using anti-inflammatory drugs raise some concern. This evidence is supported from the results of an Italian study, where the authors found that Italian general practitioners did not use ICS to treat a large number of patients with persistent asthma [42]. Nevertheless, even a poor adherence to the GINA guidelines in asthma patients must be taken into account [43].

### Limitations of the study

Some caveats should be considered in the interpretation of our results because of the impossibility to assess the level of the control of asthma through objective measures, as the baseline and follow-up surveys were based on telephone interviews [14]. Control refers to the control of the manifestations of asthma at a multidimensional level [44]. However, it is noteworthy to consider that the assessment of lung function could only have led to a worsening of asthma control. In fact, if a subject had had a normal lung function, his/her level of asthma control would have been dependent on the other markers of control used in the definition and so it would have remained unchanged. Otherwise, if a subject had had reduced lung function, his/her level of control could have remained unchanged or could have been worse. Lung function assessment is also important in the definition of remission. A more stringent definition of asthma remission could require not only a prolonged absence of symptoms, but also an objective demonstration of normal airway function and responsiveness [27]. Another limitation of the study is related to the definition of allergic rhinitis, as allergological tests were not available.

### Strengths of the study

To our knowledge, this is one of the few cohort studies evaluating asthma control according to the GINA classification over a nine-year period in a relatively large, random sample of subjects with current asthma in Italy. Furthermore, we had the opportunity to simultaneously evaluate changes in asthma control and asthma remission. Unlike the studies carried out on hospital patients or outpatients, this study was based on subjects with asthma sampled from the general population. Therefore, even subjects with mild forms of the disease could have been included in the survey.

## Conclusion

In conclusion, in the present study we found that nearly 1/3 (29.7%) of the subjects with current asthma sampled from the Italian adult population aged 20–44 years had recovered from their asthma after about 10 years, whereas only 26.3% of the subjects with active asthma showed controlled asthma. Asthma control at baseline seems to be an important predictor of asthma remission and asthma control at follow up.

Clinical asthma control is highly relevant at individual and societal levels, and measures of control (which to some extent reflect severity) are related to remission probability.

## References

[1] Bahadori K, Doyle-Waters MM, Marra C, Lynd L, Alasaly K, et al (2009) Economic burden of asthma: a systematic review. BMC Pulm Med 19: 9–24. 10.1186/1471-2466-9-24 PMC2698859 19454036

[2] de Marco R, Cappa V, Accordini S, Rava M, Antonicelli L, et al (2012) Trends in the prevalence of asthma and allergic rhinitis in Italy between 1991 and 2010. Eur Respir J 39: 883–92. 22005911 10.1183/09031936.00061611

[3] Gershon A, Guan J, Victor JC, Wang C, To T (2012) The course of asthma activity: a population study. J Allergy Clin Immunol 129: 679–86. 22178637 10.1016/j.jaci.2011.11.014

[4] Braman SS (2010) Growing old with asthma: what are the changes and challenges? Expert Rev Respir Med 4: 239–48. 20406090 10.1586/ers.10.12

[5] de Marco R, Poli A, Ferrari M, Accordini S, Giammanco G, et al (2002) The impact of climate and traffic-related NO2 on the prevalence of asthma and allergic rhinitis in Italy. Clin Exp Allergy 32: 1405–12. 12372117 10.1046/j.1365-2745.2002.01466.x

[6] Holm M, Omenaas E, Gíslason T, Svanes C, Jögi R, et al (2007) Remission of asthma: a prospective longitudinal study from northern Europe (RHINE study). Eur Respir J 30: 62–5. 17360725 10.1183/09031936.00121705

[7] Ekerljung L, Rönmark E, Larsson K, Sundblad BM, Bjerg A, et al (2008) No further increase of incidence of asthma: incidence, remission and relapse of adult asthma in Sweden. Respir Med 102: 1730–6. 18760582 10.1016/j.rmed.2008.07.011

[8] Bateman ED, Bousquet J, Busse WW, Clark TJ, Gul N, et al (2008) Stability of asthma control with regular treatment: an analysis of the Gaining Optimal Asthma controL (GOAL) study. Allergy 63: 932–8. 18588561 10.1111/j.1398-9995.2008.01724.x

[9] Taylor DR, Bateman ED, Boulet LP, Boushey HA, Busse WW, et al (2008) A new perspective on concepts of asthma severity and control. Eur Respir J 32: 545–54. 18757695 10.1183/09031936.00155307

[10] Upham JW, James AL. Remission of asthma: The next therapeutic frontier? (2011) Pharmacol Ther. 130: 38–45. 10.1016/j.pharmthera.2011.01.002 21232553

[11] Bronnimann S, Burrows B (1986) A prospective study of the natural history of asthma. Remission and relapse rates. Chest 90: 480–4. 3757559 10.1378/chest.90.4.480

[12] Rönmark E, Jönsson E, Lundbäck B (1999) Remission of asthma in the middle aged and elderly: report from the Obstructive Lung Disease in Northern Sweden study. Thorax 54: 611–13. 10377206 10.1136/thx.54.7.611 PMC1745510

[13] de Marco R, Bugiani M, Cazzoletti L, Carosso A, Accordini S, et al (2003) The control of asthma in Italy. A multicentre descriptive study on young adults with doctor diagnosed current asthma. Allergy 58: 221–8. 12653796 10.1034/j.1398-9995.2003.00059.x

[14] de Marco R, Cazzoletti L, Cerveri I, Corsico A, Bugiani M, et al (2005) Are the asthma guideline goals achieved in daily practice? A population-based study on treatment adequacy and the control of asthma. Int Arch Allergy Immunol 138: 225–34. 16210861 10.1159/000088723

[15] Global Initiative for Asthma (2006) Global strategy for asthma management and prevention. NHLBI/WHO workshop report. Bethesda (MD): National Institutes of Health. National Heart Lung and Blood Institute publication no. 02–3659.

[16] Cazzoletti L, Marcon A, Janson C, Corsico A, Jarvis D, et al (2007) Asthma control in Europe: a real-world evaluation based on an international population-based study. J Allergy Clin Immunol 120: 1360–7. 17981317 10.1016/j.jaci.2007.09.019

[17] Global Initiative for Asthma. Global strategy for asthma management and prevention 2011 update. Available at: http://www.ginasthma.org/uploads/users/files/GINA_Report_2011.pdf. Accessed January 15, 2012.

[18] Little RJA, Rubin DB (2002) Statistical Analysis with missing data. (2nd edn). New York: John Wiley & Sons, Inc.

[19] Höfler M, Pfister H, Lieb R, Wittchen HU (2005) The use of weights to account for non-response and drop-out. Soc Psychiatry Psychiatr Epidemiol 40: 291–9. 15834780 10.1007/s00127-005-0882-5

[20] Potter FJ (1990) A study of procedures to identify and trim extreme sampling weights. Proceedings of the Survey Research Methods Section of the American Statistical Association, 225–230.

[21] Elliott MR, Little RJA (2000) Model-based alternatives to trimming survey weights. J Off Stat 16: 191–209.

[22] Williams RL (2000) A note on robust variance estimation for cluster-correlated data. Biometrics 56: 645–646. 10877330 10.1111/j.0006-341x.2000.00645.x

[23] Panhuysen CI, Vonk JM, Koëter GH, Schouten JP, van Altena R, et al (1997) Adult patients may outgrow their asthma: a 25-year follow-up study. Am J Respir Crit Care Med 155: 1267–72. 9105065 10.1164/ajrccm.155.4.9105065

[24] Horak E, Lanigan A, Roberts M, Welsh L, Wilson J, et al (2003) Longitudinal study of childhood wheezy bronchitis and asthma: outcome at age 42. BMJ 326: 422–3. 12595380 10.1136/bmj.326.7386.422 PMC149441

[25] Sears MR, Greene JM, Willan AR, Wiecek EM, Taylor DR, et al (2003) A longitudinal, population-based, cohort study of childhood asthma followed to adulthood. N Engl J Med 349: 1414–22. 14534334 10.1056/NEJMoa022363

[26] Vonk JM, Postma DS, Boezen HM, Grol MH, Schouten JP, et al (2004) Childhood factors associated with asthma remission after 30 year follow up. Thorax 59: 925–9. 15516465 10.1136/thx.2003.016246 PMC1746857

[27] Broekema M, Volbeda F, Timens W, Dijkstra A, Lee NA, et al (2010) Airway eosinophilia in remission and progression of asthma: accumulation with a fast decline of FEV(1). Respir Med 104: 1254–62. 20434897 10.1016/j.rmed.2010.03.030

[28] Burgess JA, Matheson MC, Gurrin LC, Byrnes GB, Adams KS, et al (2011) Factors influencing asthma remission: a longitudinal study from childhood to middle age. Thorax 66: 508–13. 21450787 10.1136/thx.2010.146845

[29] de Marco R, Locatelli F, Cerveri I, Bugiani M, Marinoni A, et al (2002) Incidence and remission of asthma: a retrospective study on the natural history of asthma in Italy. J Allergy Clin Immunol 110: 228–35. 12170262 10.1067/mai.2002.125600

[30] Rabe KF, Adachi M, Lai CK, Soriano JB, Vermeire PA, et al (2004) Worldwide severity and control of asthma in children and adults: the global asthma insights and reality surveys. J Allergy Clin Immunol 114: 40–7. 15241342 10.1016/j.jaci.2004.04.042

[31] Peters SP, Jones CA, Haselkorn T, Mink DR, Valacer DJ, et al (2007) Real-world Evaluation of Asthma Control and Treatment (REACT): findings from a national Web-based survey. J Allergy Clin Immunol 119: 1454–61. 17481716 10.1016/j.jaci.2007.03.022

[32] Demoly P, Annunziata K, Gubba E, Adamek L (2012) Repeated cross-sectional survey of patient-reported asthma control in Europe in the past 5 years. Eur Respir Rev 21: 66–74. 22379176 10.1183/09059180.00008111 PMC9487469

[33] Combescure C, Chanez P, Saint-Pierre P, Daurès JP, Proudhon H, et al (2003) Assessment of variations in control of asthma over time. Eur Respir J 22: 298–304. 12952264 10.1183/09031936.03.00081102

[34] Koster ES, Raaijmakers JA, Vijverberg SJ, Koenderman L, Postma DS, et al (2011) Limited agreement between current and long-term asthma control in children: the PACMAN cohort study. Pediatr Allergy Immunol 22: 776–83. 21749459 10.1111/j.1399-3038.2011.01188.x

[35] Siroux V, Boudier A, Bousquet J, Bresson JL, Cracowski JL, et al (2009) Phenotypic determinants of uncontrolled asthma. Allergy Clin Immunol 124: 681–7. 10.1016/j.jaci.2009.06.010 19665764

[36] Bottema RW, Reijmerink NE, Koppelman GH, Kerkhof M, Postma DS (2005) Phenotype definition, age, andgender in the genetics of asthma and atopy. Immunol Allergy Clin North Am 25: 621–639. 16257629 10.1016/j.iac.2005.07.002

[37] Leynaert B, Bousquet J, Henry C, Liard R, Neukirch F (1997) Is bronchial hyperresponsiveness more frequent in women than in men? A population-based study. Am J Respir Crit Care Med 156: 1413–1420. 9372654 10.1164/ajrccm.156.5.9701060

[38] Lee JH, Haselkorn T, Chipps BE, Miller DP, Wenzel SE (2006) Gender differences in IgE-mediated allergic asthma in the epidemiology and natural history of asthma: Outcomes and Treatment Regimens (TENOR) study. J Asthma 43: 179–184. 16754518 10.1080/02770900600566405

[39] Braido F, Baiardini I, Balestracci S, Ghiglione V, Stagi E, et al (2009) Does asthma control correlate with quality of life related to upper and lower airway real life study. Allergy 64: 937–943. 19243359 10.1111/j.1398-9995.2008.01932.x

[40] Chauhuri R, McSharry C, McCoard A, Livingston E, Hothersall E, et al (2008) Role of symptoms and lung function in determining asthma control in smokers with asthma. Allergy 63: 132–135. 18053022 10.1111/j.1398-9995.2007.01538.x

[41] Ponte EV, Franco R, Nascimento HF, Souza-Machado A, Cunha S, et al (2008) Lack of control of severe asthma is associated with co-existence of moderate-to-severe rhinitis. Allergy 63: 564–9. 18394130 10.1111/j.1398-9995.2007.01624.x

[42] Baldacci S, Maio S, Simoni M, Cerrai S, Sarno G, et al (2012) The ARGA study with general practitioners: impact of medical education on asthma/rhinitis management. Respir Med 106: 777–85. 22436656 10.1016/j.rmed.2012.02.013

[43] Corsico AG, Cazzoletti L, de Marco R, Janson C, Jarvis D, et al (2007) Factors affecting adherence to asthma treatment in an international cohort of young and middle-aged adults. Respir Med 101: 1363–7. 17188854 10.1016/j.rmed.2006.11.012

[44] Lundbäck B, Dahl R (2007) Assessment of asthma control and its impact on optimal treatment strategy. Allergy 62: 611–9. 17508964 10.1111/j.1398-9995.2007.01399.x

